# The role and molecular mechanism of D-aspartic acid in the release and synthesis of LH and testosterone in humans and rats

**DOI:** 10.1186/1477-7827-7-120

**Published:** 2009-10-27

**Authors:** Enza Topo, Andrea Soricelli, Antimo D'Aniello, Salvatore Ronsini, Gemma D'Aniello

**Affiliations:** 1 Stazione Zoologica Anton Dohrn, 80121, Villa Comunale, 80121, Napoli, Italy; 2 Università di Napoli Parthenope e Fondazione IRCCS-SDN, Via Gianturco 113, 80143 Naples, Italy; 3 Department of Obstetrics and Gynecology, and Reproductive Medicine (IVF Unit), Hospital 'S Luca', 84078 Vallo della Lucania, Italy

## Abstract

**Background:**

D-aspartic acid is an amino acid present in neuroendocrine tissues of invertebrates and vertebrates, including rats and humans. Here we investigated the effect of this amino acid on the release of LH and testosterone in the serum of humans and rats. Furthermore, we investigated the role of D-aspartate in the synthesis of LH and testosterone in the pituitary and testes of rats, and the molecular mechanisms by which this amino acid triggers its action.

**Methods:**

For humans: A group of 23 men were given a daily dose of D-aspartate (DADAVIT^®^) for 12 days, whereas another group of 20 men were given a placebo. For rats: A group of 10 rats drank a solution of either 20 mM D-aspartate or a placebo for 12 days. Then LH and testosterone accumulation was determined in the serum and D-aspartate accumulation in tissues. The effects of D-aspartate on the synthesis of LH and testosterone were gauged on isolated rat pituitary and Leydig cells. Tissues were incubated with D-aspartate, and then the concentration (synthesis) of LH and cGMP in the pituitary and of testosterone and cAMP in the Leydig cells was determined.

**Results:**

In humans and rats, sodium D-aspartate induces an enhancement of LH and testosterone release. In the rat pituitary, sodium D-aspartate increases the release and synthesis of LH through the involvement of cGMP as a second messenger, whereas in rat testis Leydig cells, it increases the synthesis and release of testosterone and cAMP is implicated as second messenger. In the pituitary and in testes D-Asp is synthesized by a D-aspartate racemase which convert L-Asp into D-Asp. The pituitary and testes possesses a high capacity to trapping circulating D-Asp from hexogen or endogen sources.

**Conclusion:**

D-aspartic acid is a physiological amino acid occurring principally in the pituitary gland and testes and has a role in the regulation of the release and synthesis of LH and testosterone in humans and rats.

## Background

D-Aspartic acid (D-Asp) is an endogenous amino acid which has been found in the neuroendocrine tissues of both invertebrates and vertebrates [1]. D-Asp was first found in the nervous system of marine mollusks [2] and subsequently in the nervous and endocrine tissues of many other animals, including humans [1]. High levels of D-Asp occur transiently in the brain of chickens, rats and humans during the last stage of embryonic life, suggesting that it has a role in the development of the nervous system [3-6]. In addition, within the nervous system this amino acid is concentrated in the axon terminals (synaptosomes) and in synaptic vesicles together with L-Asp and L-Glu [7]; additionally, it is involved in visual activity [8], suggesting it has a role in neurotransmission.

In the endocrine system, high concentrations of D-Asp have been recorded in rat testes at birth as well as following sexual maturity [5]. Further research involving rats showed the highest concentrations of D-Asp in testicular venous blood plasma, as well as in the rete testis, the epididimus, testicular parenchymal cells, seminiferous tubules, interstitial fluid and spermatozoa [9], suggesting that D-Asp is involved in spermatogenesis. A specific D-Asp localization was further observed in rat testes either in elongate spermatidits [10] or in Leydig cells [11]. Several further studies have demonstrated that D-Asp is concentrated in the endocrine gland, particularly in the pineal gland, the pituitary and the testis [1,12]. It has been observed that D-Asp in rats is capable of eliciting the release of the gonadotropin-releasing hormone (GnRH) from the hypothalamus, the luteinizing hormone (LH) and the growth hormone (GH) from the pituitary gland, and testosterone from the testes [13]. In addition, D-Asp occurs in a high concentration in the pineal gland [14], where it modulates melatonin synthesis in rat pinealocytes [15], and is implicated in the α-melanocyte-stimulating hormone, GABA, and in dopamine release [16]. In sheep, D-Asp is endogenously present in tissues and is electively stored in endocrine glands, such as the pituitary, and in the brain after its administration. NMDA and LH increased following D-Asp administration [17]. Recently an *in vitro *study carried out on boar testes revealed that endogenous testicular D-Asp enhances gonad aromatase activity [18], the key enzyme that converts testosterone into 17β-estradiol. In addition, studies done on the testes and ovary of the lizard *Podarcis s. sicula *have shown similar findings, confirming that D-Asp is involved in the local production of estrogen [19,20].

On the basis of the above findings, D-Asp seems to play a crucial role in reproduction either due to its suggested role of neuromodulator or because it is involved in biosynthesis and the release of sexual hormones. Recent studies analyzed the role of D-Asp in human reproduction in both females and males. In men, a lower D-Asp content was found in oligoasthenoteratospermic seminal fluid and spermatozoon, and a relationship exists between the amount and motility of semen and the content of D-Asp [21]. In women, it has been found that D-Asp occurs in the follicular fluid as a physiological component, and interestingly, the concentration of D-Asp in the fluid is reduced in older women. In addition, the concentration of D-Asp in the follicular fluid is lower, as are the quality of the oocytes and the level of fertilization [22].

Although numerous studies have been conducted on this matter, no investigations have been done until now on the effects of D-Asp on the secretion of LH and testosterone in humans, and neither has the molecular mechanism by which D-Asp triggers it action in the synthesis and release of these hormones been investigated. This study aims to evaluate the effects of D-aspartate administration on LH and testosterone production in humans and rats and to understand the biochemical mechanisms by which D-Asp induces the synthesis and release of LH and testosterone, using rats as the model animal.

## Methods

### Determination of D-Asp by HPLC associate with D-AspO

The determination of D-Asp in serum and tissues was carried out using an HPLC enzymatic method combined with D-aspartate oxidase (D-AspO) according to our previously described method [8]. For the serum, 0.4 ml serum was mixed with 3.6 ml of 0.2 M TCA and centrifuged at 10,000 g for 10 min. The supernatant was purified on cation exchange resin (AG 50WX8). The purified sample was dissolved in 0.4 ml H_2_O and then 50 μl were used for HPLC according to the described method [8]. For solid tissues 10-100 mg tissues were homogenized at a ratio of 1:50 with 0.1 M TCA and centrifuged as above. The supernatant was purified as above and analyzed at HPLC as described [8]. In order to quantify the content in amino acids in the sample, a standard curve consisting of 50 pmoles of D-Asp and 100 pmoles of each of the following amino acids: L-Asp, L-Glu, L-Ser, L-Thr, L-His, Gly, L-Arg, L-Ala, L-Val, L-Met, L-Tyr, L-Phe, L-Ile, L-Leu and L-Lys was carried out in the same assay condition. The method allowed the determination with high reliability of a minimum amount of D-Asp at a coefficient of variation (CV) of 5% as calculated from 10 repeated analyses of D-Asp from a sample in which a known amount of D-Asp has been added.

### Effects of D-aspartate on LH and testosterone in serum release in humans and rats

The experiment using human subjects was carried out on two groups of healthy male volunteers aged between 27 and 37 years at the IVF (in vitro fertilization) Unit, Hospital "S. Luca", Vallo della Lucania, Italy. The first group was composed of 23 volunteers who constituted the experimental group; the second group was composed of 20 volunteer who constituted the placebo group. Informed consent was obtained from each participant and the procedure was approved by the Institutional Review Board of the Hospital. Every morning at breakfast for 12 consecutive days subjects in the first group were invited to consume, by mouth a solution of 10 ml of 2.0 M sodium D-aspartate (3.12 g/10 ml) supplemented with vitamin B6, folic acid and vitamin B12 and diluted in half a glass of water or fruit juice. This solution is marketed in Italy under the name DADAVIT^® ^(Pharmaguida s.r.l., Italy) and used as a supplement to increase the quality of human seminal fluid. The participants belonging to the placebo group received 10 ml of a solution consisting of 2 M NaCl (1.12 g/10 ml in water) containing the same vitamins as above, and this solution was packaged as DADAVIT^® ^solution (termed DADAVIT^® ^placebo). Blood samples (8-10 ml) from each participant were taken before treatment, after 6 days of treatment, after 12 days of treatment, and 3 days after suspension of the treatment. The blood was taken in the morning between 9.30 and 10.30 a.m., a time when serum oscillations of LH and testosterone are at their minimum value [23]. The determination of LH and testosterone in human blood was carried out using commercially available immunochemoluminescence kits for LH and testosterone purchased from Bayer HealthCare LLC, Subsidiary System (Bayer Corporation, USA). According to the manufacturing company that have prepared the kits for LH and testosterone, the sensitivity of the method was 0.1 mIU/ml for LH and 0.2 ng/ml for testosterone.

The experiments on rats were carried out as follows: Adult Wistar male rats (120 days old, 340 ± 20 g), purchased from Charles River laboratory (Italy), were housed 2 per cage in a controlled environmental animal facility with a 12-h light/dark cycle and fed with laboratory food pellets and water *ad libitum *until experimentation. All the procedures involving rats were in accordance with institutional guidelines. When ready, three groups of 10 animals each were prepared. Rats in the first group were allowed to drink a solution consisting of 20 mM of sodium D-aspartate for 12 days and then sacrificed. Rats in the second group were given to drink a solution of 20 mM sodium D-aspartate to drink for 12 consecutive days; then allowed to drink tap water for 3 days and then sacrificed. Rats in of the third group (control) were given to drink a solution of 20 mM NaCl to drink for 12 days and then sacrificed. After rats were sacrificed, the blood and solid tissues (frontal cortex, hippocampus, pituitary, testis, liver and kidney) were collected. The serum was obtained from the blood (after coagulation and centrifugation at 3,000 g for 30 min) and the concentration of LH and testosterone (hormone release) determined. The solid tissues were homogenized in PBS at a ratio of 1:20 and centrifuged at 10,000 g for 30 min. The supernatant was used for the analysis of D-Asp by HPLC.

The determination of LH in the rat serum was carried out using a reagent kit from Amersham Biosciences (EIA Biotrak™ System RPN 2562; Amersham Biosciences Europe GmbH, Cologno Monzese, Milano, Italy), whereas the concentration of testosterone in the rat serum was determined using the same kits and the same procedure that were used for the human serum.

### Effects of D-aspartate on the rat pituitary in the synthesis of LH and cGMP: In vitro experiments

The pituitary was taken from an adult male rat (120 days old) and cut longitudinally into two portions, and then incubated with moderate shaking at 37°C for 60 min in 1.0 ml of Krebs-Ringer solution, pH 7.4, containing 50 μl of a cocktail of protease inhibitors (Sigma, P 8340) and also contained 1 mg/ml BSA in air/CO_2 _(19:1) and D-Asp at the concentration of 0.1 mM (10 μl of 10 mM). The same experiment was repeated using a second male rat, but D-Asp was at the concentration of 1.0 mM (10 μl of 100 mM). The same experiment was repeated on a third male rat, but without D-Asp (control). After incubation, each sample was homogenized in its incubation solution and divided into two equal portions. The first portion was centrifuged at 10,000 rpm for 5 min at 4°C, and the supernatant was used for LH determination (synthesis). The second portion was mixed with 25 μl of 1 M HCl and 25 μl of 1 M TCA, then homogenized and centrifuged. The supernatant was neutralized with 1 M NaOH and used for the determination of cGMP (cyclic guanosine monophosphate; 3',5'-cyclic guanosine monophosphate) and cAMP (cyclic adenosine monophosphate; 3',5'-cyclic adenosine monophosphate). These determinations were carried out using a radioimmunoassay based on the competition between unlabelled cAMP or cGMP and a fixed quantity of the tritium labeled compound (Amersham Biosciences, Buckinghamshire, England (cAMP: code TRK432; cGMP: code TRK500). This experiment was repeated five times on sample from different animals.

### Effects of D-aspartate on rat Leydig cells in the synthesis of testosterone and cAMP: In vitro experiments

Leydig cells were prepared from the testes of 5 rats according to the described procedure [24]. The purified Leydig cells were suspended at a concentration of 1 × 10^6 ^cells/ml in Krebs-Ringer solution containing a cocktail of protease inhibitors (Sigma, P 8340, diluted 1:100) and BSA 1 mg/ml. To 1 ml of this suspension were added, respectively, 10 μl of 10 mM of sodium D-aspartate and 10 μl of 100 mM of sodium D-aspartate (0.1 and 1.0 mM, respectively). For the control, 10 μl of H_2_O was used instead of D-Asp. The samples were incubated for 60 min at 37°C with moderate shaking. Then each sample was homogenized in its solution and divided into two equal portions. The first portion was centrifuged at 10,000 g for 5 min, and the supernatant was used for the testosterone determination. The second portion was mixed with 50 μl of 1 M HCl and 50 μl of 1 M TCA, then homogenized and centrifuged, and the supernatant was used for the determination of cAMP and cGMP. This experiment was repeated five independent times.

### Biosynthesis of D-aspartate in rat tissues: D-aspartate racemase activity

The endogenous presence of D-Asp in rat tissues and in particular in the pituitary gland and testis has suggested that this amino acid is synthesized *in vivo *by an aspartate racemase (EC 5.1.1.13) which converts L-Asp to D-Asp. This enzyme has been found in bacteria [25] in mollusks [7,8,26], in amphibians [19], and in rat neurons [6]. In this study we determined the activity of this enzyme that we have termed 'D-Aspartate racemase because it converts L-Asp into D-Asp, using a modified procedure of the described method [7]. The procedure consists of two steps: i) incubation of the sample with L-Asp to generate D-Asp; and ii) determination of D-Asp by D-AspO with a colorimetric method. The reactions involved in the entire procedure are:



Step 1: Rat tissues were homogenized at a ratio of 1:10 in 0.1 M Tris-HCl, pH 8.0, containing 10 mM EDTA, and centrifuged at 20,000 g for 20 min. Then, 500 μl of the supernatant was mixed with 500 μl of 0.2 M sodium L-aspartate and incubated at 37°C for 60 min. A blank was prepared as a sample, but incubated at 0°C (ice water) instead of 37°C, for 60 min. After incubation, 200 μl of 1.0 M TCA was added, and the solution was mixed and centrifuged for 10 min at 20,000 g.

Step 2: 1 ml of each supernatant (sample and blank) was neutralized with 80-90 μl of 2.0 M NaOH and mixed with 100 μl Tris-HCl 1 M, pH 8.2 and 5 μl of purified D-AspO (2 mg/ml; 300 U/ml), obtained by over-expression from beef kidney [8] or from the hepathopancreas of *Octopus vulgaris *[2] and incubated for 30 min at 37°C in order to oxidize the D-Asp in α-oxaloacetate. After that, 100 μl of 5.0 mM 2,4-dinithrophenylhydrazine (in 5 M HCl) was added, mixed and left at room temperature for 10 min. Finally, 200 μl of 5 M NaOH was added and mixed, and the absorbance of the sample was read against the blank at 445 nm. In order to determine the amount of D-Asp in the 1 ml of the above supernatant, a standard test of D-Asp was carried out. For this purpose, 1.0 ml of 0.01 mM of sodium D-aspartate was mixed with 100 μl of H_2_O, 100 μl Tris-HCl 1 M, pH 8.2 and 5 μl of purified D-AspO and the procedure was continued as sample. The absorbance of this standard was read against a blank in which 1.0 ml H_2_O was used instead of D-aspartate. One enzyme unity (EU) was defined as the amount of the enzyme capable of generating 1 nmol of D-Asp in 60 min of incubation at 37°C in the above assay conditions. The specific activity was defined as the EU/mg of the homogenate.

### Statistical analysis

Results are presented as the mean ± SEM. LH and testosterone concentrations in human serum were analyzed by analysis of variance with repeated measurements (ANOVA, StatView). D-Asp storage in rat tissues and LH and testosterone concentration from *in vivo *and *in vitro *experiments on rats were analyzed by one-way ANOVA. Pairwise comparison of the means was made with Fisher's LSD test. Values of *p *< 0.05 were considered significant.

## Results

### Specific determination of D-aspartic acid by HPLC-D-AspO

**Figure 1:**
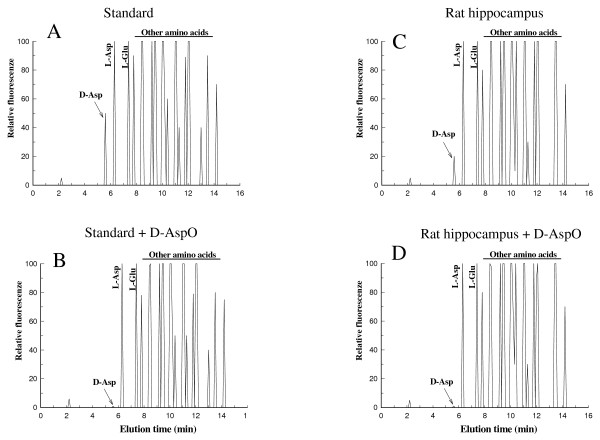
**Typical HPLC analysis of D-Asp and other amino acids from rat pituitary gland**. Panel A, HPLC profile of amino acids from a standard mixture consisting of 50 pmoles of D-Asp and 100 pmoles of various L-amino acids. Panel B is the HPLC analysis of the same amino a cids, but after treatment with D-AspO. Panel C shows the HPLC analysis of free amino acids from 0.05 mg of rat hippocampus, and panel D is the same sample, but after treatment with D-AspO. The peak of D-Asp disappeared completely after incubation of the sample with D-AspO (panels B and D), indicating that the peak area of D-Asp was completely due to D-Asp.

The determination of free D-Asp in the sample was carried out using an HPLC method associated with the use of D-aspartate oxidase (D-AspO), as previously reported [8]. Fig. 1 shows a typical HPLC analysis of a standard mixture of amino acids and a biological sample. Panel A shows the HPLC analysis of a mixture consisting of 20 pmoles of D-Asp and 50 pmoles of various L-amino acids. Panel B, shows the same HPLC analysis of the amino acid standard mixture, but after treatment with D-AspO. Panel C shows the HPLC analysis of free amino acids from 0.05 mg of a rat hippocampus; and panel D shows the same sample, but after treatment with D-AspO. This figure shows that in both the standard mixture (panel B) and the sample (panel D) the peak of D-Asp disappeared completely after the incubation of these samples with D-AspO, indicating that the peak of D-Asp was completely due to D-Asp in the standard mixture or in the sample, respectively.

### Effects of D-aspartate on LH and testosterone release in humans

In this study 23 participants took an oral dose of sodium D-aspartate (DADAVIT^®^) for 12 consecutive days and 20 participants took an oral dose of placebo (DADAVIT^® ^placebo) for 12 consecutive days; the levels of LH and testosterone in the serum were monitored after 6 and 12 days of treatment and 3 days after suspension of the treatment (with D-aspartate or the placebo).

Concerning the LH pattern, the results demonstrated that after 12 days of D-Asp treatment, 20 out of 23 (87%) participants had significantly increased concentrations of LH in their blood with respect to basal values (the value of LH found in the same subjects before starting treatment). Statistical analysis demonstrated that the value (mean ± SEM) of serum calculated for all the 23 subjects treated with D-Asp increased by 33.3%. From a basal-level mean of 4.2 ± 0.5 mIU/ml, LH rose to a mean value of 5.6 ± 0.9 mIU/ml (Table 1). The increase was statistically significant (ANOVA with repeated measures: [F_(2,82) _= 24.279, p < 0.0001]). LH concentration determined in the placebo group after D-Asp treatment compared with the level before treatment had not increased [ANOVA: F_(1,82) _= 0.643, p > 0.427]), thus indicating that the increase of LH due to D-aspartate treatment was authentic. The effect of D-aspartate on LH increase was time dependent. When subjects drank sodium D-aspartate for 6 days, LH increased only 1.07-fold, and this value was not statistically significant (Table 1). However, when the treatment of D-Asp was continued for 12 consecutive days, the LH concentration in the serum increased significantly (benefit effects). In order to know how long LH remained increased in the blood after the suspension of the treatment, we measured the concentration of LH in the serum 3 days after the D-Asp treatment or the placebo treatment was suspended. The results indicated that 3 days after suspension of D-Asp treatment, LH was still found at a 1.14-fold increased levels compared with the respect of basal level, but not statistically significant (Table 1).

**Table 1:** Effects of D-aspartate on LH and testosterone release in human serum

	**Basal levels treatment**	**After 6 days of D-Asp treatment**	**After 12 days of D-Asp**	**3 days after the suspension of D-Asp**
	**LH **(mIU/ml serum)			
Subjects treated with with Na-D-aspartate	4.2 ± 0.5	4.5 ± 0.6	5.6 ± 0.9*	4.8 ± 0.8
Subjects treated with placebo	4.2 ± 0.4	4.3 ± 0.7	4.2 ± 0.6	4.1 ± 0.5
				
	**Testosterone **(ng/ml serum)			
Subjects treated with Na-D-aspartate	4.5 ± 0.6	5.2 ± 0.7	6.4 ± 0.8*	5.8 ± 0.6*
Subjects treated with placebo	4.6 ± 0.5	4.5 ± 0.7	4.7 ± 0.7	4.6 ± 0.7

These results are the mean ± SEM of LH and testosterone in serum of 23 participants (aged 27-37 years) at zero time (basal levels), after 6 days and 12 days of D-aspartate treatment and 3 days after the suspension of treatment. Subjects drank a daily dose of DADAVIT^® ^(Na-D-aspartate). The placebo treatment was carried out on another 20 participants of the same age, who drank a daily dose of DADAVIT^® ^placebo. Statistical analysis was conducted using ANOVA with repeated measures (post-hoc test). * Values are statistically significant for p < 0.001.

Concerning the effect of D-Asp on the induction of testosterone release, after 12 days of D-Asp treatment, the levels of testosterone in the serum of the participants were significantly increased compared with basal levels. Out of 23 participants, 20 had increased testosterone. From a mean of 4.5 ± 0.6 ng/ml serum at zero time, it rose to 6.4 ± 0.8 ng/ml, a 42% increase (Table 1). Statistical analyses indicated a significant effect [ANOVA with repeated measures: treatment effect: F_(1,82) _= 7.724, p < 0.0082] and a significant interaction between treatment and days [F_(2,82) _= 32.599; P < 0.0001]. As with LH, so also with testosterone, the effect of D-aspartate was time dependent. When subjects were treated with sodium-D-aspartate for only 6 days, testosterone was found of 1.15-fold higher than basal levels, but this increase was not statistically significant (Table 1). Interestingly 3 days after the suspension of D-Asp treatment, testosterone was still increased 1.22-fold compared with the basal levels (5.8 ± 0.6 ng/ml against 4.5 ± 0.6 ng/ml). Fisher's post-hoc analysis also revealed a significant difference in the testosterone concentration in the serum 3 days after the end of the treatment (p < 0.01) (Table 1). One plausible explanation of this phenomenon is that since in rats ingested D-Asp remains accumulated in the testes in significant amounts until 3 days after the suspension of D-Asp treatment (see below), if it is assumed that in humans D-Asp also remains significantly increased in the testes 3 days after the suspension of D-Asp treatment, we can deduce that in humans as in rats, D-Asp had remained accumulated in significant amounts in the testes and consequently it continued to stimulate testosterone release.

### Effects of D-aspartate on the release of LH and testosterone in rats

When rats drank a solution of 20 mM sodium D-aspartate for 12 days, the concentration of LH and testosterone in the serum increased significantly. After treatment, the level of LH in the rats' serum was increased by 51% compared with that of the controls. From a mean LH value of 3.7 ± 0.3 mIU/ml for the controls, it increased to a value of 5.6 ± 0.4 mIU/ml, a 1.51-fold increase (p < 0.001) (Table 2). Three days after the suspension of the treatment, LH concentration still remained increased compared with the control rats but the difference was not statistically significant (Table 2).

**Table 2:** Effects of D-aspartate on LH and testosterone release in rat serum

	**Rats treated with placebo**	**Rats treated with Na-D-Aspartate for 12 days**	**Rat treated with Na-D-Asp for 12 days and then for 3 days with placebo**
**LH** (mIU/ml serum)	3.7 ± 0.3	5.6 ± 0.4*	4.1 ± 0.3
**Testosterone** (ng/ml serum)	5.1 ± 0.4	10.4 ± 1.2*	6.1 ± 0.5*

A group of 10 male rats (120-130 days old) were allowed to drink a solution of 20 mM NaCl. A second group of rats were allowed to drink a solution of 20 mM of Na-D-aspartate for 12 days and a third group of rats were allowed to drink 20 mM Na-D-aspartate for 12 days and then tap water for 3 days. After treatment each rat was sacrificed and LH and testosterone in serum determined. Values are the mean ± SEM). Asterisks indicate that the difference in the value between treated rats and control rats was significant at p < 0.001, evaluated by one-way ANOVA.

Concerning testosterone, the effect of sodium D-aspartate on testosterone release in rat serum was to induce a 2.05-fold increase of this hormone; from a basal level of 5.1 ± 0.8 ng/ml in the control rats, testosterone reach the level of 10.4 ± 1.2 ng/ml in treated animals (p < 0.001) (Table 2). It is interesting to observe that, contrary to what occurred with LH, three days after the suspension of the treatment, testosterone levels still remained significantly increased compared with the control rats: a 1.27-fold increase (p < 0.01) (Table 2).

The effect of D-Asp on the increased release of testosterone in serum that occurs 3 days after suspension of the treatment may be due to two events: i) the increase of testosterone in serum is a consequence of the increased LH, which in turn stimulates the release of testosterone in the testes; and ii) D-Asp also has a direct action on the testes in enhancing testosterone release, as already demonstrated both previously [5,13] and also by this study (see below). In addition, since the ingested D-Asp also still remains accumulated significantly in the testes 3 days after D-Asp suspension of treatment (this study), we deduce that the persistent increase of testosterone in rat blood is due to the accumulation of D-Asp that persists leading to stimulated testosterone production.

### Occurrence and accumulation of D-aspartate in rat tissues following D-Asp treatment

The results obtained from this experiment demonstrated that D-Asp occurs naturally at a comparatively higher concentration in the pituitary gland, where the mean concentration was found to be 129 ± 12 nmol/g tissue (Table 3), followed by the testes (109 ± 8 nmol/g tissue), followed by other tissues (Table 3). After treatment with D-Asp for 12 days, D-Asp was found to be accumulated in all the rat tissues examined (Table 3). However, the pituitary, the testes and the thyroid were the tissues in which this amino acid was accumulated in the greatest amounts. In the pituitary, in fact, D-Asp was increased 6.8-fold compared with the basal levels (Table 2). In the testes D-Asp was increased 7.15-fold compared within to the basal levels. However we found that in all rat tissues 12 hours after from D-Asp treatment, D-Asp was found accumulated at a level that was 2-3 times greater than the basal levels (Table 3).

**Table 3:** Endogenous occurrence of D-aspartate in rat tissues and accumulation after treatment with sodium D-aspartate

	**Basal levels of D-Asp**	**Levels of D-Asp after 12 days of D-Asp treatment**	**Levels of D-Asp 3 days after the suspension of D-Asp treatment**
Pituitary	129 ± 12	880 ± 45*	145 ± 12
Testes	109 ± 8	780 ± 45*	205 ± 14*
Thyroid	90 ± 6	350 ± 19*	110 ± 9
Hippocampus	62 ± 5	108 ± 9*	78 ± 7
Frontal cortex	41 ± 4	85 ± 7*	55 ± 5
Liver	12 ± 3	24 ± 2*	11 ± 3
Kidney	10 ± 2	45 ± 4*	13 ± 3
Serum	4 ± 1	8 ± 2*	3 ± 1

A group of 10 male rats (120-130 days old) rats were allowed to drink 20 mM sodium D-aspartate for 12 days. A second group of 10 male rats of the same age were allowed to drink 20 mM sodium D-aspartate for 12 days and then drink tap water for 3 days. A third group of 10 rats male rats of the same age drank 20 mM of NaCl for 12 days (placebo). All rat were sacrificed and collected serum and solid tissues and determined the concentration of D-Asp. Values are the mean ± SEM. Asterisks indicated that the difference between treated animals and control were statistically significant for a p < 0.001 evaluated by one-way ANOVA.

### Effects of D-aspartate on the synthesis of LH and cGMP in the isolated rat pituitary

**Figure 2:**
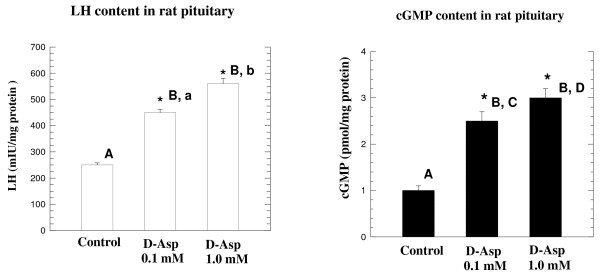
**Effects of D-Asp on LH and cGMP synthesis in isolated rat pituitary glands**. Pituitary glands were incubated at 37°C for 60 min in a medium with Na-D-aspartate 0.1 or 1.0 mM and then LH and cGMP levels were determined in the total gland homogenate plus medium. The left panel shows that the increase of LH (LH synthesis) due to the action of the two concentrations of D-Asp versus the control (pituitary incubated without D-Asp): A, B (p < 0.001); a, b (p < 0.02). The right panel shows the total concentration of cGMP determined in the same samples as above obtained (cGMP synthesis): A, B and C, D(p < 0.001). Results are the mean ± SEM obtained from 5 individual experiments. Asterisks indicate that the difference in value between treated rats and control rats was significant at p < 0.0001, evaluated by one-way ANOVA.

*In vitro *experiments carried out on the isolated pituitary gland demonstrated that when the gland is incubated with 0.1 mM D-Asp, a significantly increased (1.8-fold) the synthesis of LH occurs. In fact, whereas the control sample contained an LH value of 250 ± 8 mIU/mg protein, in the sample incubated with D-Asp, the LH concentration rose to 480 ± 11.4 mIU/mg, p < 0.001) (Fig. 2, left panel). The same effects occurred also when the concentration of D-Asp in the medium was 1.0 mM. In this case the increased LH synthesis was enhanced 2.32-fold (from 250 ± 8 mIU/mg of protein to 580 ± 19.7 mIU/mg) (A, B: p < 0.001; a, b: p < 0.02), but the increase was not proportionate to the concentration of D-Asp (Fig. 2, left panel). In this experiment we also measured the concentration of cAMP and cGMP in the medium in which the pituitary gland was incubated with D-Asp and found that the concentration of cGMP was significantly increased. A concentration of 0.1 mM D-Asp in 60 min of incubation induced a significant increase in the synthesis of cGMP it raised 2.5-fold, from a value of 1.0 ± 0.2 pmol/mg tissue to 2.5 ± 0.4 pmol/mg tissue (Fig. 2, right panel). D-Asp at the concentration of 1.0 mM in the medium induced a 3.1-fold increase in the synthesis of cGMP (A, B and C, D: p < 0.0001) (Fig. 2, right panel). However, the increase was not proportional to D-Asp concentration, thus indicating that the minimum concentration of 0.1 mM is already sufficient to stimulate the pituitary to synthesize cAMP; this is similar to the effects of other described molecules on the pituitary gland [27-29].

### Effects of D-aspartate on the synthesis of testosterone and cAMP in rat Leydig cells

**Figure 3:**
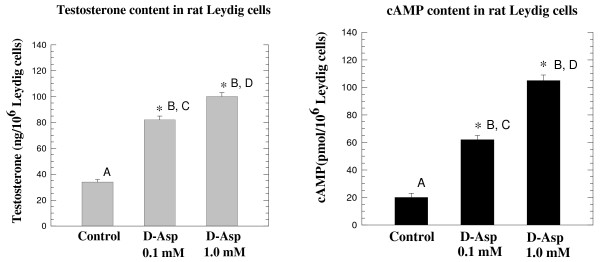
**Effects of D-Asp on the synthesis of testosterone and cAMP in isolated rat Leydig cells**. 1 ml of purified Leydig cells (1.0 × 10^6 ^cells) was incubated at 37°C for 60 min with Na-D-Asp 0.1 or 1.0 mM and control and then the testosterone and cAMP synthesis was determined. The left panel shows the total production of testosterone in the medium plus the Leydig cell homogenate, in the control sample and in the samples incubated with 0.1 mM or with 1.0 mM D-Asp: A, B and C, D (p < 0.001). The right panel shows the concentration of cAMP in the same above samples: A, B and C, D (p < 0.001). Values are the mean ± SEM obtained from 5 individual experiments. Asterisks indicate that the increased levels were significant compared with the controls at p < 0.001 as evaluated by one-way ANOVA.

When Leydig cells obtained from rat testes were incubated with 0.1 mM D-Asp, there was a significant 2.4-fold increase in the synthesis of testosterone. From a basal value of 34 ± 3 ng testosterone/10^6 ^in Leydig cells, after D-Asp treatment, testosterone was raised to 82 ± 3 ng/10^6 ^cells (A, B: p < 0.001) (Fig. 3, left panel). When the concentration of D-Asp was 1.0 mM in the medium, the increase was 2.94-fold compared with the control (C, D: p < 0.001) (Fig. 3, left panel). Thus, as occurred with LH synthesis in the pituitary gland, in Leydig cells the concentration of 0.1 mM D-Asp also induced testosterone synthesis significantly. We looked for the action of D-Asp on the second messenger and found that following treatment of Leydig cells with 0.1 mM D-Asp, cAMP was increased 3.1-fold compared with the control. When D-Asp was administered at a levels of 0.1 mM in the medium, from a value of 20 ± 3.0 pmol/10^6 ^in Leydig cells in the control, cAMP rose to 62 ± 8.1 pmol/10^6 ^(p < 0.001) (Fig. 3, right panel). When D-Asp was administered at a level of 1.0 mM in the medium, a 5.25-fold increase was observed (20 ± 3.0 pmol/10^6 ^compared with 105 ± 3.3 pmol/10^6 ^after stimulation) (p < 0.0001) (Fig. 3, right panel). These results thus mirrored the previously reported data in which it was demonstrated that the stimulation of Leydig cells is accompanied by an increase in cAMP [30].

### Biosynthesis of D-aspartate in rat tissues: D-aspartate racemase

**Figure 4:**
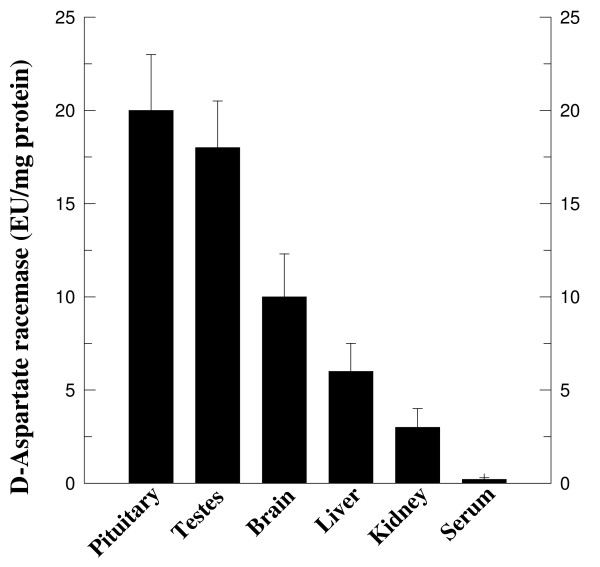
**D-Aspartate racemase activity in rat tissues: Synthesis of D-Asp**. Homogenized tissue were incubated with L-Asp for 60 min at 37°C and then the amount of D-Asp synthesized was detected. The D-Asp that developed was determined by a colorimetric method based on the use of D-AspO. One enzyme unity (EU) was defined as the amount of the enzyme capable of generating 1 nmol of D-Asp in 60 min of incubation at 37°C and in the above assay conditions. The specific activity was defined as the EU/mg protein. The values are the mean ± SEM as obtained from five determinations, each carried out on a different rat.

The presence of D-Asp in rat tissues leads to the question of the origin of D-Asp. In order to determine whether D-Asp is biosynthesized *in vivo *by conversion of L-Asp to D-Asp, we incubated a sample homogenized with L-Asp and then measured the D-Asp generated. We have termed the enzyme which converts L-Asp into D-Asp "D-aspartate racemase". The results obtained from this investigation have demonstrated that this enzyme is present in many rat tissues, and that of the investigated tissues (pituitary, testes, brain, liver, kidney and serum), the pituitary gland is the tissue which contains the highest enzymatic activity, with 20 ± 3.0 EU/mg protein, followed by: testis; brain, liver, kidney and serum (Fig. 4). In addition, it is also interesting to observe that the D-aspartate racemase activity is greater in those tissues in which D-Asp also occurs at the higher levels.

## Discussion

In this study using a specific HPLC method combined with the use of D-AspO (Fig. 1) and specific immunoenzymatic methods for the determination of LH and testosterone as well as of the second messengers, cAMP and cGMP, we have demonstrated that D-aspartic acid plays a role in the release and synthesis of LH and testosterone in humans and rats. In humans we found that with the consumption of a daily dose of 10 ml of 2 M sodium D-aspartate solution (3.12 g) for 12 consecutive days, the levels of LH and testosterone in the serum were significantly increased, by 33% and 42% respectively. In 87% of the subjects who have been treated with sodium D-aspartate increased the concentration of LH and testosterone in the serum (p < 0.0001). After 6 days of treatment LH was already found to have increased, but this increase was not statistically significant (Table 1). Three days after sodium D-aspartate suspension, LH still was found higher than that of basal levels (a 1.14-fold increased), but this increase was not statistically significant (Table 1), indicating that the increase of LH was dose dependent. The consumption of sodium D-aspartate in humans also induced significant testosterone release in the serum. In fact, in the same subjects who took D-Asp for 12 days, the levels of testosterone increased significantly. ANOVA with repeated measurements indicated a significant value (p < 0.0001). As with LH, so also with testosterone, 6 days of treatment induced increased serum testosterone in the blood, but this increase was not significant. Interestingly, contrary to what occurred for LH, three days after suspension of the treatment, testosterone still remained increased in the serum 1.28-fold increase compared with the basal levels, and this increase was significant (p < 0.01) (Table 1). A possible explanation of this event is that the ingested D-Asp probably also remained accumulated in the testes 3 days after treatment was stopped, and it continued to stimulate the testosterone production in the testes. This hypothesis is supported by results obtained in rats. In fact, after rats had drunk a solution of 20 mM D-Asp for 12 days, this amino acid had accumulated in various tissues, especially the pituitary and the testes (Table 3). However, when D-Asp treatment was suspended, D-Asp that had accumulated in tissues diminished until it reached basal levels, except in the testes, where D-Asp still remained significantly increased 1.88 fold (Table 3).

In this study we used rats as model animals in order to understand the molecular mechanism by which D-Asp induce its action. Rats were allowed to drink a solution of sodium D-Asp for 12 days and after that the concentration of D-Asp that accumulated in tissues was determined along with the concentration of LH and testosterone. In rat as in humans, D-Asp also actively induced LH and testosterone release. In fact, when rats were treated with D-Asp, a significant increase in LH and testosterone was observed after the 12 days of treatment (Table 2), coinciding with the increased levels of D-Asp in the pituitary and testis respectively (Table 2). Thus, these data indicate that D-Asp is involved in the regulation of the above hormones.

*In vitro *experiments conducted on an isolated rat pituitary incubated with D-Asp demonstrated that D-Asp at the concentration of 0.1 mM in the medium is capable of inducing the synthesis of LH (Fig. 2, left panel) and testosterone (Fig. 3, left panel). In addition, when the pituitary gland was incubated with 0.1 mM D-Asp, a significant increase of cGMP occurred in the assay mixture (Fig 3, right panel) thus indicating that the release and synthesis of LH occurred under the intervention of the cGMP. These data are in agreement with previous results obtained by other investigators [27-29], who demonstrated that the molecule involved in the signal transduction for other metabolites in the rat pituitary was cGMP. Similarly, *in vitro *experiments conducted on isolated Leydig cells incubated in a medium containing 0.1 mM of sodium D-Asp demonstrated that this amino acid is capable of inducing the synthesis of testosterone (Fig. 3, left panel) and that this event is mediated by cAMP, which acts as the molecule involved in the signal transduction for testosterone synthesis from rat Leydig cells (Fig. 3, right panel). Also, this last result is in agreement with previous studies by other investigators in which it was demonstrated that the increase of testosterone synthesized by Leydig cells occurs under the intervention of the cAMP [30]. The final concern of this study was to examine the biosynthesis of D-Asp in the pituitary and testis. The results demonstrated that rat tissues contain a racemase activity that is capable of converting L-Asp into D-Asp. We have termed this enzyme "D-aspartate racemase" and it is present in all the rat tissues we analyzed. The pituitary and the testis are the tissues with higher concentrations (Fig. 4). These data thus indicate that a relationship exists between the endogenous concentration of D-Asp and the concentration of the D-aspartate racemase in the tissues (Fig. 4).

In this study, we also investigated the action of L-Asp on hormone release in rats. A group of 10 male rats were treated with L-Asp instead of D-Asp at the same time and at the same concentration and then levels of LH and testosterone in the blood were determined. The results of this investigation indicated that the L-Asp does not induce any significant increase of serum LH or testosterone (data not shown), thus indicating thus that only the stereochemical form of D-Asp is active in the hormone release.

## Conclusion

Here we demonstrated that D-aspartic acid, which occurs as a physiological compound in the mammalian pituitary and testis, has a role in the regulation of the release and synthesis of LH and testosterone. In humans and rats, sodium D-Asp treatment enhances the release of LH and testosterone. The experiments that we carried out on rats have permitted us to understand that this amino acid regulates the synthesis of LH and testosterone in the pituitary and the testis respectively. This action is mediated in the pituitary by cGMP and in the testis by cAMP, which act as the second messengers in the signal transduction in the pituitary and testes respectively. The pituitary and testis possesses a D-Aspartate racemase, which provides the necessary production of D-Asp.

## Abbreviations

(LH): Luteinizing hormone; (D-Asp): D-aspartic acid; (HPLC): High performance liquid chromatography; (TCA): trichloroacetic acid; (IVF): in vitro fertilization; (DADAVIT^®^): Commercial integrator consisting of sodium D-aspartate and vitamins, produced by the 'Pharmaguida s.r.l., Italy'; (cGMP): cyclic guanosine monophosphate; 3',5'-cyclic guanosine monophosphate; (cAMP): cyclic adenosine monophosphate; 3',5'-cyclic adenosine; (PBS): phosphate buffer saline.

## Competing interests

The authors declare that they have no competing interests.

## Authors' contributions

ET and ADA participated in amino acids analysis by HPLC, treatment of rats with sodium D-Asp and placebo, determination of hormone analysis, preparation of rat pituitary slices and Leydig cells, analysis of cAMP, cGMP and racemase activity at the Zoological Station of Naples. AS participated in the determination of hormones in serum at the Fondazione IRCCS-SDN). SR and GDA oversaw recruitment of human subjects (volunteers) at the Department of Reproductive Medicine (IVF Unit), Hospital 'S. Luca', Vallo della Lucania, Italy). GDA, ET and ADA designed the experiments and prepared the manuscript. All authors have read and approved the final manuscript.

## References

[B1] D'Aniello A (2007). D-Aspartic acid: An endogenous amino acid with an important neuroendocrine role. Brain Res Rev.

[B2] D'Aniello A, Giuditta A (1977). Identification of D-aspartic acid in the brain of *Octopus vulgaris*. J Neurochem.

[B3] Neidle A, Dunlop DS (1990). Developmental changes of free D-aspartic acid in the chicken embryo and in the neonatal rat. Life Sci.

[B4] Hashimoto A, Kumashiro S, Nishikawa T, Oka T, Takahashi K, Mito T, Takashima S, Doi N, Mizutani Y, Kaneco T, Ootomo E (1993). Embryonic development and postnatal changes in free D-aspartate and D-serine in the human prefrontal cortex. J Neurochem.

[B5] D'Aniello A, Di Cosmo A, Di Cristo C, Annunziato L, Petrucelli L, Fisher GH (1996). Involvement of D-aspartic acid in the synthesis of testosterone in rat testes. Life Sci.

[B6] Wolosker A, D'Aniello A, Snyder SH (2000). D-aspartate disposition in neuronal and endocrine tissues: ontogeny, biosynthesis and release. Neuroscience.

[B7] Spinelli P, Brown E, Ferrandino G, Branno M, Montarolo PG, D'Aniello E, Rastogi RK, D'Aniello B, Baccari G, Fisher G, D'Aniello A (2006). D-Aspartic acid in the nervous system of *Aplysia limacine*: Possible Role in Neurotransmission. J Cell Physiol.

[B8] D'Aniello S, Spinelli P, Ferrandino G, Peterson K, Tsesarskia M, Fisher GH, D'Aniello A (2005). Cephalopod vision involves dicarboxylic amino acids: D-aspartate, L-aspartate and L-glutamate. Biochem J.

[B9] D'Aniello A, Di Fiore MM, D'Aniello G, Colin FE, Lewis G, Setchell BP (1998). Secretion of D-aspartic acid by the rat testis and its role in endocrinology of the testis and spermatogenesis. FEBS Letters.

[B10] Sakai K, Homma H, Fukushima JA, Santa T, Tashiro T, Iwatsubo K, Imai K (1998). Localization of D-aspartic acid in elongate spermatids in rat testis. Arch Biochem Biophys.

[B11] Nagata Y, Homma H, Lee JA, Imai K (1999). D-Aspartate stimulation of testosterone synthesis in rat Leydig cells. FEBS Letters.

[B12] Takemtsu F, Homa H (2005). Free D-aspartate in Mammals. Biol Pharm Bull.

[B13] D'Aniello A, Di Fiore MM, Fisher GH, Milone A, Seleni A, D'Aniello S, Perna A, Ingrosso D (2000). Occurrence of D-Aspartic acid and N-methyl-D-aspartic acid in rat neuroendocrine tissues and their role in the modulation of luteinizing hormone and growth hormone release. FASEB J.

[B14] Imai K, Fukushima T, Hagiwara K, Santa T (1995). Occurrence of D-aspartic acid in rat brain and pineal gland. Biomed Chromatogr.

[B15] Ishio S, Yamada H, Hayashi M, Yatsushiro S, Noumi T, Yamaguchi A, Moriyama Y (1998). D-Aspartate modulates melatonin synthesis in rat pinealocytes. Neurosci Lett.

[B16] Pampillo M, Scimonelli T, Bottin MC, Duvilanski BH, Rettori V, Seilicovich A, Lasaga M (2002). The effect of D-aspartate on luteinizing hormone-releasing hormone, α-melanocyte-stimulating hormone, GABA and dopamine release. Neuroreport.

[B17] Boni R, Santillo R, Macchia G, Spinelli P, Ferrandino G, D'Aniello A (2006). D-Aspartate and reproductive activity in sheep. Theriogenology.

[B18] Lamanna C, Assisi L, Botte V, Di Fiore MM (2007). Involvement of D-Asp in P450 aromatase activity and estrogen receptors in boar testis. Amino acids.

[B19] Raucci F, D'Aniello S, Di Fiore MM (2005). Endocrine role of D-aspartic acid in the testes of lizard *Podarcis s. sicula*. J Endocrinol.

[B20] Assisi L, Botte V, D'Aniello A, Di Fiore MM (2001). Enhancement of aromatase activity by D-aspartic acid in the ovary of the lizard *Podarcis s. sicula*. Reproduction.

[B21] D'Aniello G, Ronsini S, Guida F, Spinelli P, D'Aniello A (2005). Occurrence of D-aspartic acid in human spermatozoa: Possible role in reproduction. Fertil Steril.

[B22] D'Aniello G, Grieco N, Di Filippo MA, Cappiello F, Topo E, D'Aniello E, Ronsini S (2007). Reproductive implication of D-aspartic acid in human pre-ovulatory follicular fluid. Human Reprod.

[B23] Wilson JD, Bondy PK, Rosenberg LE (1980). The testis. Metabolic control and Disease.

[B24] Vasta V, Shimizu-Albergine M, Beavo JA (2006). Modulation of Leydig Cell function by cyclic nucleotide phospfodiesterase 8A. Proc Natl Acad Sci USA.

[B25] Yohda M, Okada H, Kumagai H (1991). Molecular cloning and nucleotide sequencing of the aspartate racemase gene from lactic acid bacteria *Streptococcus thermophilus*. Biochem Biophys Acta.

[B26] Shibata K, Watanabe T, Yoshikawa H, Abe K, Takahashi S, Kera Y, Yamada RH (2003). Purification and characterization of aspartate racemase from the bivalve mollusk *Scapharca broughtonii*. Comp Biochem Physiol B.

[B27] Snyder G, Naor Z, Fawcett CP, McCann SM (1980). Gonadotropin release and cyclic nucleotides: Evidence for luteinizing hormone-releasing hormone induced elevation of guanosine 3',5'-monophosphate levels in gonadotrophs. Endocrinology.

[B28] Kawakami M, Kimura F (1980). Stimulation of guanosine 3',5'-monophosphate accumulation in anterior pituitary glands in vivo by synthetic luteinizing hormone-releasing hormone. Endocrinology.

[B29] Garrel G, Lozach A, Bachir LK, Laverriere JN, Counis R (2002). Pituitary adenylate cyclase-activating polypeptide stimulates nitric-oxide synthase type I expression and potentiates the cGMP response to gonadotropin releasing hormone of rat pituitary gonadotrophs. J Biol Chem.

[B30] Saez JM (1994). Leydig cells: endocrine, paracrine, and autocrine regulation. Endocr Rev.

